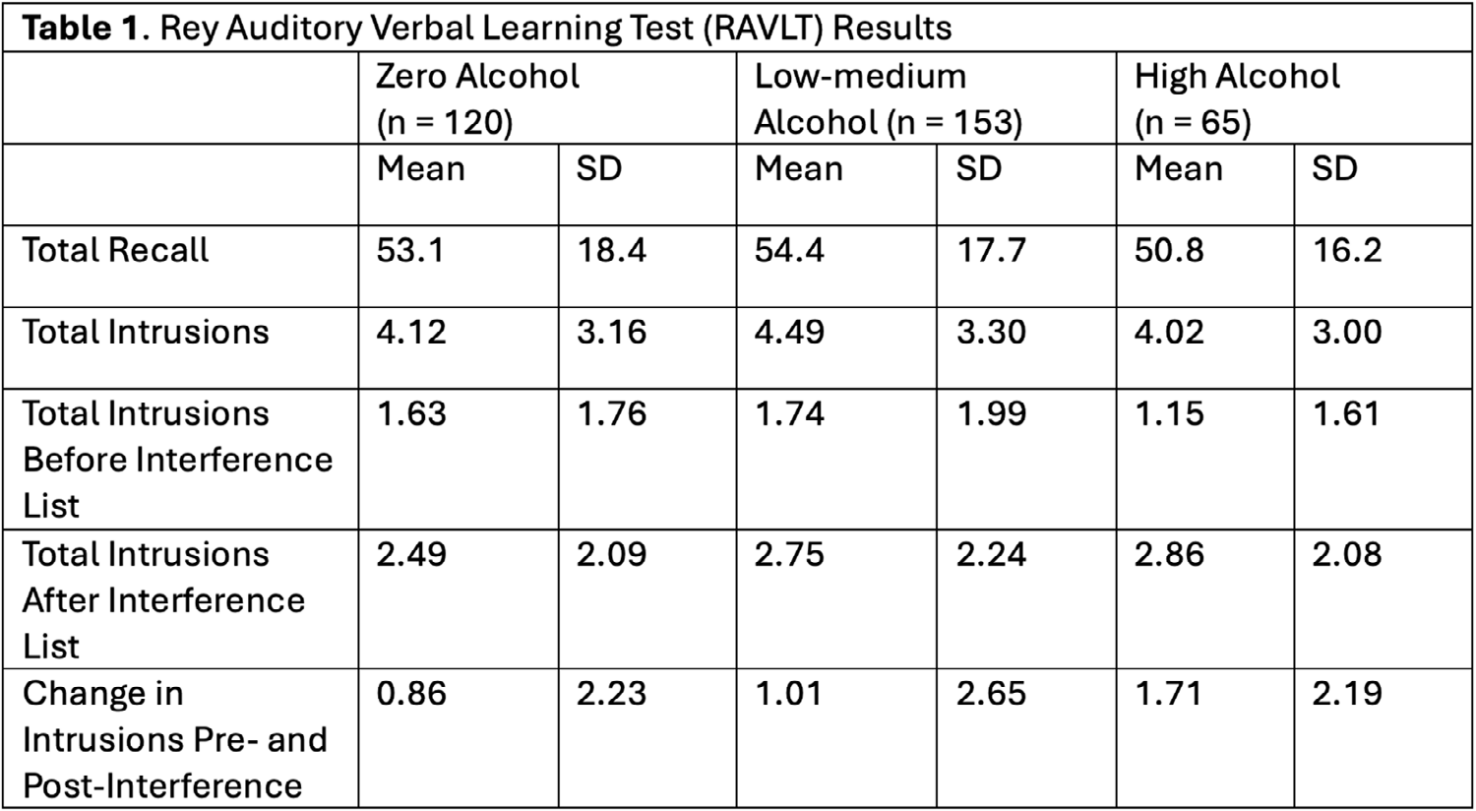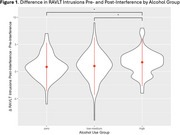# Examining the Effects of Excessive Alcohol Use on Verbal Memory Processes in Older Adults with Mild Cognitive Impairment

**DOI:** 10.1002/alz70857_105860

**Published:** 2025-12-26

**Authors:** Ari Cuperfain, Sara Pishdadian, Janine Louis, Malcolm A Binns, Sandra E. Black, Howard Chertkow, Morris Freedman, Clement Ma, Mario Masellis, Paula M McLaughlin, Joel Ramirez, David F. Tang‐Wai, Carmela Tartaglia, Sanjeev Kumar, the CCNA COMPASS‐ND Group, the ONDRI Group

**Affiliations:** ^1^ University of Toronto Temerty Faculty of Medicine, Toronto, ON, Canada; ^2^ Centre for Addiction and Mental Health, Toronto, ON, Canada; ^3^ Rotman Research Institute, Baycrest Health Sciences, Toronto, ON, Canada; ^4^ Sunnybrook Health Sciences Centre, University of Toronto, Toronto, ON, Canada; ^5^ Jewish General Hospital, McGill University, Montreal, QC, Canada; ^6^ Sunnybrook Health Sciences Centre, Toronto, ON, Canada; ^7^ Nova Scotia Health, Halifax, NS, Canada; ^8^ Memory Clinic, Toronto Western Hospital, University Health Network, Toronto, ON, Canada

## Abstract

**Background:**

Excessive alcohol use (EAU) is associated with cognitive impairment. Previously we have shown that older adults with EAU have higher rates of intrusions during verbal memory recall. Here, we further characterize the effects of alcohol on verbal memory processes by examining different types of intrusions during recall.

**Method:**

Two multicenter cohorts, the Comprehensive Assessment of Neurodegeneration and Dementia and the Ontario Neurodegenerative Disease Research Initiative, provided data for this study. Participants were diagnosed with MCI due to Alzheimer's disease (AD‐MCI) or cerebro‐vascular disease (V‐MCI) per established criteria, and categorized based on their current and past alcohol use into ‘zero’, ‘low‐medium’ (less than 1 to 7 standard drinks/week), or ‘high’ (>7 standard drinks/week) alcohol use groups. We coded the intrusions on the English version of the Rey Auditory Verbal Learning Test (RAVLT) as those with or without interference, i.e. before or after presentation of list B during list A recall. We calculated the change in intrusions with and without interference and used independent t‐tests to compare this change between high/zero, high/low‐medium, and low‐medium/zero alcohol groups.

**Result:**

We included 338 participants with mean (SD) age = 71.62 (7.58) years. The zero alcohol group included 120 (females = 50.0%; V‐MCI = 55.8%), the low‐medium alcohol group 153 (females = 40.5%; V‐MCI = 47.7%), and the high alcohol group 65 (females = 21.5%; V‐MCI = 50.8%) participants. There were no differences in total or specific intrusion types between the groups. Participants in the high alcohol group exhibited greater increase in intrusions, mean (SD) = 1.71 (2.19), versus 1.01 (2.65) in the low‐medium group, and 0.86 (2.23) in the zero group following exposure to the interference list. Differences were significant for comparisons between high/zero (*p* = 0.014) and high/low‐medium groups (*p* = 0.047), but not between low‐medium/zero groups (*p* = 0.60).

**Conclusion:**

Participants with MCI and EAU are more susceptible to interference from recently learned task‐irrelevant information during recall, suggestive of impaired cognitive control. These findings may inform future research regarding cognitive profiles and cognitive training interventions for older adults with MCI with EAU.